# Non-Coding Regulatory Variants in Autoimmune Disease: Biological Mechanisms, Immune Context, and Integrative Multi-Omics Interpretation

**DOI:** 10.3390/biology15050407

**Published:** 2026-02-28

**Authors:** Ahmed S. A. Ali Agha, Nawras A. Al-Zaki, Saif Aldeen Nasser Alshammari, Lama Odeh, Renata Obekh, Nour Sameer, Hussam M. Askari, Nancy Hakooz, Ibrahim Al-Adham, Phillip J. Collier

**Affiliations:** 1 Department of Pharmaceutical Sciences, School of Pharmacy, The University of Jordan, Amman 11942, Jordan; norlightscholz@gmail.com; 2 Fachbereich Chemie & Biologie, Hochschule Fresenius, Limburger Straße 2, 65510 Idstein, Germany; al-shammari.saif@stud.hs-fresenius.de; 3 Department of Biology and Biotechnology, Faculty of Science, The Hashemite University, Zarqa 13133, Jordan; lamaodeh739@gmail.com (L.O.); renata.931931@gmail.com (R.O.); 4 School of Medicine, Jordan University of Science and Technology, Irbid 22110, Jordan; hmaskari20@med.just.edu.jo; 5 Department of Biopharmaceutics and Clinical Pharmacy, School of Pharmacy, University of Jordan, Amman 11942, Jordan; nhakooz@ju.edu.jo; 6 Faculty of Pharmacy and Medical Sciences, University of Petra, Amman 11196, Jordan; ialadham@uop.edu.jo (I.A.-A.); pip.collier@yahoo.com (P.J.C.)

**Keywords:** autoimmune disease, non-coding regulatory variants, immune regulation, genome-wide association studies, single-cell eQTL mapping, spatial transcriptomics, multi-omics integration

## Abstract

Autoimmune diseases arise from complex interactions between genetic susceptibility, immune regulation, and tissue-specific inflammatory processes, yet most risk variants identified by genome-wide association studies occur in non-coding regions with poorly defined biological functions. This review addresses the challenge of interpreting non-coding regulatory variants in autoimmunity by synthesizing emerging analytical frameworks that integrate functional genomics, single-cell profiling, spatial transcriptomics, and multi-omics data. We describe stepwise strategies that refine statistical associations through regulatory annotation, immune cell–state resolution, and perturbational evidence, highlighting complementary approaches such as massively parallel reporter assays, transcriptome-wide association studies, and single-cell expression quantitative trait locus mapping. These methods demonstrate that many autoimmune risk variants exert context-dependent effects that emerge only in specific immune cell states, activation trajectories, or tissue microenvironments. Advances in spatial and chromatin-informed technologies further clarify how regulatory variation shapes immune circuits in diseases such as systemic lupus erythematosus and rheumatoid arthritis. Finally, we discuss how machine learning-enabled multi-omics integration supports molecular endotyping and therapeutic inference while emphasizing interpretability and reproducibility. Collectively, this review highlights a shift from static variant annotation toward dynamic, context-aware analytical frameworks that enable mechanism-informed interpretation of genetic risk in autoimmune disease.

## Simple Summary

Autoimmune diseases such as systemic lupus erythematosus and rheumatoid arthritis arise when the immune system attacks the body’s own tissues. Large genetic studies have found many DNA changes linked to these diseases, but most of these changes occur outside genes, in regions that control when and where genes are switched on. Because these “control” regions work differently depending on the immune cell type, its activation state, and the affected tissue, it is often difficult to explain how a risk variant contributes to disease. In this review, we describe a step-by-step approach to interpret non-gene DNA variants in autoimmunity by combining genetic signals with evidence from gene regulation maps, single-cell profiling, and tissue-level spatial studies. We summarize how newer methods can reveal which immune cells and inflammatory conditions expose the effects of risk variants, and how laboratory tests can confirm whether a variant truly changes gene activity. Finally, we explain how combining multiple biological data types with artificial intelligence can help define disease subtypes, improve risk prediction, and suggest drug repurposing opportunities. Together, these strategies support more accurate, mechanism-based understanding of autoimmune disease and can guide precision diagnosis and targeted treatment.

## 1. Introduction

Autoimmune diseases are characterized by multi-layered complexity, involving genetic susceptibility, dysregulated immune signaling, and tissue-specific inflammatory responses [1]. Genome-wide association studies (GWAS) have identified hundreds of risk loci across conditions such as systemic lupus erythematosus (SLE) and rheumatoid arthritis (RA), yet over 75% of these variants reside in non-coding regions, limiting their interpretability within conventional analytical frameworks [2].

In this review, the term “non-coding regulatory variants” primarily refers to cis-regulatory DNA variants that influence gene expression without altering protein-coding sequence. These include variants located in enhancers, promoters, insulators, and other regulatory elements that modulate transcriptional activity, enhancer–promoter interactions, or chromatin accessibility [3]. Where explicitly indicated, the term is used more broadly to include additional non-coding regulatory mechanisms, such as variants affecting splicing regulation or endogenous retroviral elements (HERVs) with enhancer-like activity. Variants that alter protein-coding sequence are outside the scope of this review unless their primary effect is regulatory.

Single-cell and multi-omic profiling now resolve immune cell states, activation trajectories, and regulatory networks with granularity inaccessible to bulk approaches. Mechanistic linkage of non-coding variation to effector programs, immune stratification, and therapeutic targets remains incomplete, largely due to fragmented analytical integration across regulatory layers [4].

The objective of this review is to synthesize emerging analytical strategies that resolve regulatory complexity in autoimmune disease beyond conventional variant-centric approaches. The review describes the shift from static annotation pipelines to modular, context-aware frameworks that integrate functional genomics, pseudotime-resolved eQTL mapping, spatial transcriptomics, and chromatin-informed TWAS. The review also discusses how multi-omics integration and machine learning are increasingly enabling predictive modeling, molecular endotyping, and therapeutic inference. The review outlines key methodological developments and describes a unified analytical architecture that reconstructs regulatory logic across dynamic immune states and spatial contexts.

From a translational perspective, the key challenge is not the availability of analytical tools, but their meaningful integration. Clinically actionable insight requires identifying the immune cell states and tissue contexts in which non-coding risk variants are functional and linking these effects to effector genes or regulatory modules that define molecular endotypes. Importantly, these endotypes must improve diagnostic, prognostic, or therapeutic decision-making beyond standard clinical and serologic measures. For this reason, the present review prioritizes integrative analytical logic over individual methodologies, focusing on their translational relevance.

**Figure 1:**
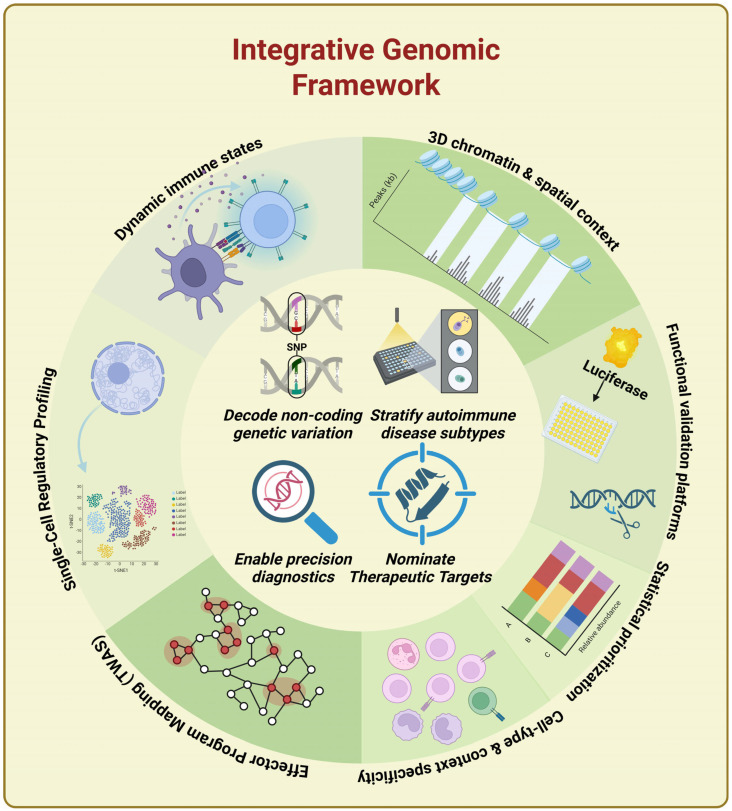
A modular analytical architecture linking non-coding genetic variation to immune dysfunction through integrated platforms. Key components include dynamic immune-state modeling, 3D chromatin and spatial context, functional validation (e.g., MPRA, luciferase), single-cell regulatory profiling, TWAS-based effector mapping, statistical prioritization, and cell-type/context-specific resolution. Together, these modules enable mechanistic disease stratification, precision diagnostics, and therapeutic target nomination.

Emphasis is placed on analytical strategies with demonstrated mechanistic relevance and translational maturity rather than exhaustive methodological cataloguing. This conceptual framework is summarized in Figure 1, which illustrates the modular components and integrative logic underlying the framework described in this review.

## 2. Analytical Challenges in Autoimmune Pathogenesis

**Figure 2:**
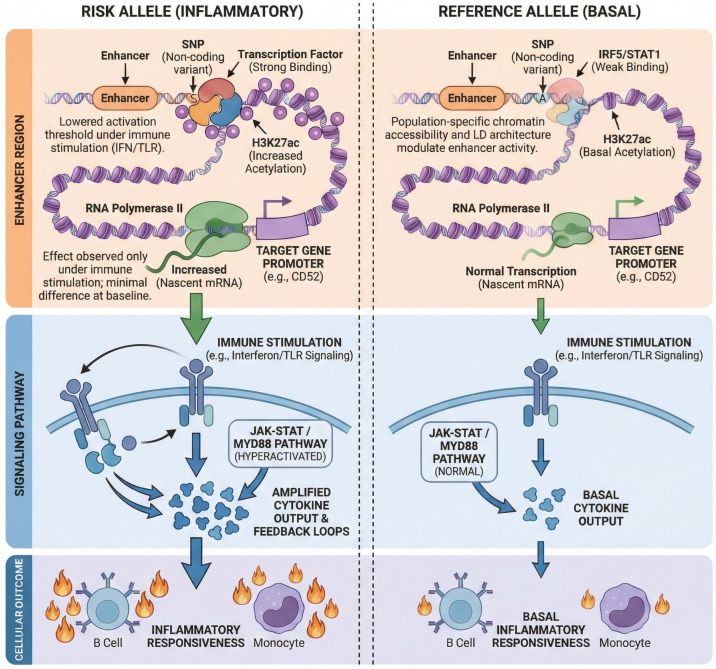
Mechanistic model of enhancer-mediated activation-threshold tuning in autoimmune risk. Schematic illustrating how non-coding variants in enhancers modulate transcription-factor binding (interferon regulatory factor 5, IRF5; signal transducer and activator of transcription 1, STAT1) and histone acetylation (histone H3 lysine 27 acetylation, H3K27ac), altering chromatin accessibility and promoter looping under immune stimulation (interferon, IFN; Toll-like receptor, TLR). In the risk allele, stronger binding increases RNA polymerase II (Pol II) recruitment and nascent transcription of target genes such as CD52, hyperactivating Janus kinase–signal transducer and activator of transcription (JAK–STAT) and myeloid differentiation primary response 88 (MYD88) signaling and amplifying cytokine output. The reference allele maintains basal enhancer activity and normal transcriptional responsiveness. This activation-threshold-tuning mechanism links population-specific chromatin accessibility and linkage disequilibrium (LD) architecture to inflammatory immune phenotypes. Genes, transcription factors, and signaling pathways shown are representative, literature-supported examples intended to illustrate a general regulatory principle rather than a locus-specific or newly derived mechanism.

Most autoimmune-associated non-coding variants do not exert constitutive effects; instead, they modulate inducible immune responses in a context-dependent manner. Functional and perturbational studies demonstrate that many autoimmune-associated variants lower activation thresholds or amplify inducible transcription rather than altering basal gene expression. This regulatory logic, summarized schematically in Figure 2, provides a unifying mechanistic framework for interpreting non-coding risk across populations and immune contexts.

Autoimmune pathogenesis emerges from the convergence of inherited risk, epigenetic regulation, immune-cell circuit dynamics, and environmental microbial inputs. Contemporary multi-ancestry GWAS, single-cell eQTL atlases, and integrative multi-omics now connect these layers mechanistically, clarifying disease initiation, progression, and heterogeneity [2,5,6].

### 2.1. Genetic Risk Interpretation Across Populations

High-throughput functional genomics platforms are increasingly used to characterize regulatory variant activity in immune-relevant contexts. Over 330 GWAS-identified SLE loci have been reported, with approximately 76–81% residing in enhancers or transcription factor-binding elements rather than protein-coding regions [2]. To functionally validate these non-coding loci, the technique of Massively Parallel Reporter Assays (MPRA) has been employed by Lu et al. (2021) and Fu et al. (2024) [7,8], to assess allele-specific enhancer activity under stimulus-specific conditions. Complementary approaches—including chromatin immunoprecipitation followed by qPCR (ChIP–qPCR), luciferase reporter assays, and CRISPR/Cas9 genome editing—have been used by Singh et al. (2021) to interrogate regulatory activity, histone modifications (e.g., H3K27ac), and causal enhancer–gene relationships [9].

These data indicate that regulatory variant effects often emerge only under immune stimulation, consistent with activation-threshold tuning.

Beyond locus discovery, fine-mapping and colocalization approaches are commonly applied to refine causal variant sets [10,11,12].

At this stage, the size and posterior mass of the credible set determine whether variants can be prioritized for focused functional interrogation or require parallel screening approaches. This prioritization step is critical for distinguishing variants likely to influence stimulus-dependent regulatory responses from those that remain statistically associated but biologically unresolved.

When LD blocks remain large—particularly in regions with ancestry-specific haplotypes—functional follow-up should prioritize assays that can test multiple linked candidates in parallel (e.g., MPRA), rather than committing early to a single “lead SNP.” Conversely, when fine-mapping yields a compact credible set, downstream steps can focus on directional regulatory evidence (allele-specific accessibility/expression, motif disruption, and stimulus-dependent enhancer activity) to distinguish causal from merely correlated variants.

Promoter–enhancer linking frameworks, such as the activity-by-contact (ABC) model [13] or promoter-capture Hi-C [14], help constrain target genes, facilitating downstream MPRA/CRISPR validation and clarifying how non-coding risk propagates through immune signaling axes. Because enhancer–gene maps are probabilistic and tissue/state dependent [15], discordance between ABC predictions, promoter-capture Hi-C loops, and eQTL/TWAS gene nominations should be treated as informative rather than as “errors” [16]. Reconciliation across enhancer–gene linking strategies is most informative when evidence derives from disease-relevant immune cell states and stimulation contexts [17]. Convergent support across orthogonal modalities, including three-dimensional chromatin contacts, chromatin activity, and expression association, further strengthens effector gene nomination [18]. Importantly, loci for which the nominated target gene shifts across cell states or contexts should not be dismissed, as such context-dependent gene regulation is a recurring feature of pleiotropic autoimmune risk [15].

Integrative resources such as PLINK [19], Open Targets [20], and Enrichr [21] enable prioritized variants to be linked to effector genes and downstream programs, including JAK–STAT and TLR–MYD88 signaling [2], while reducing circular interpretation driven by gene density rather than causality.

Collectively, these analytical strategies address key challenges in the interpretability of non-coding GWAS loci, forming a modular framework for extracting mechanistically grounded biomarkers and defining functional patient stratification units.

Mechanistic resolution of non-coding risk further depends on establishing regulatory directionality, namely whether a variant enhances, attenuates, or rewires transcriptional responses. Allele-specific enhancer assays and CRISPR-based perturbations have shown that many autoimmune-associated variants act by lowering activation thresholds or prolonging inflammatory signaling, rather than by inducing constitutive gene expression.

Regulatory directionality reflects the signed effect of a variant along a stimulus–response axis, encompassing enhancer gain, attenuation, or transcriptional rewiring. Making this definition explicit clarifies what each assay contributes (e.g., MPRA for allele-specific enhancer output; caQTL/hQTL for chromatin directionality; CRISPR perturbation for causal sign in the endogenous locus) [22,23].

This distinction is critical, as threshold-shifting variants can drive pathology only under immune stimulation, explaining their context-dependent penetrance and variable clinical expressivity.

To address the limited discovery power in disease-specific GWAS, Multi-Trait Analysis of GWAS (MTAG) has been used to increase power across genetically correlated traits [24,25]. As demonstrated in a large-scale, multi-ancestry meta-analysis of autoimmune diseases [5]. MTAG was applied across 12 cohorts and 10 related traits, identifying 16 novel SLE loci. To ensure robustness, the same study employed Replicability Analysis of Trait-Associated Signals (RATES), which models the Posterior Probability of Replicability (PPR) at each locus. This analytical framework enabled high-confidence prioritization of associations (PPR > 0.90), overcoming a longstanding challenge in reproducible signal detection. A key caveat for multi-trait approaches is that increased power can come at the cost of trait-specific interpretability [26]; therefore, follow-up steps should explicitly test whether nominated loci colocalize with SLE-relevant regulatory signals (e.g., monocyte/B-cell eQTLs) rather than assuming shared-trait discoveries are SLE-specific by default.

To resolve the functional impact of non-coding loci, the technique of Transcriptome-Wide Association Studies (TWAS), using TESLA (a chromatin-informed TWAS model) and PUMICE (Prediction Using Models Informed by Chromatin conformations and Epigenomics), should be applied. These methods integrate three-dimensional (3D) chromatin architecture and epigenomic features into gene expression imputation. In the same study [5], these tools were used to link disease-associated variants to regulatory targets, such as CD52 and UBASH3A, specifically within non-classical monocytes and B cell subsets. This demonstrated how TWAS can overcome the gap between variant-level signals and downstream effector genes by leveraging regulatory priors and chromatin topology.

However, TWAS associations remain vulnerable to LD confounding and model portability constraints across ancestry and tissue [27]; therefore, combining TWAS with explicit colocalization (e.g., PP4 thresholds) and context-matched eQTL references strengthens the causal interpretation and prevents over-interpretation of expression-imputation artifacts.

Major transcriptome-wide association study models used in autoimmune genetics are summarized in Table 1.

In translational settings, these models are most informative when paired with colocalization and context-matched eQTL references, because portability and LD confounding determine whether gene nominations can be taken forward for validation or therapeutic targeting.

To identify potential therapeutic interventions from transcriptomic data, the analytical technique of Connectivity Map (CMap) can be employed. In the study as mentioned earlier, TWAS signatures were queried against a large reference library of compound-induced gene expression profiles. By selecting compounds with transcriptomic reversal scores (τ < −75), the authors nominated several repurposing candidates, including histone deacetylase (HDAC) and mTOR inhibitors. This illustrates how analytical drug-repurposing pipelines can systematically translate gene dysregulation into pharmacological insight.

Finally, for stratifying patients based on genetic risk, the Polygenic Risk Score (PRS) method, implemented via Least Absolute Shrinkage and Selection Operator Summary Statistics (LASSOSUM), have been used [24]. In this application, PRS models integrated with clinical autoantibody markers (ANA, anti-dsDNA) achieved an area under the curve (AUC) of ~0.75. This shows how analytical modeling pipelines can enhance early-stage diagnosis and risk prediction in autoimmune settings when combined with real-world clinical data [5]. A practical translational consideration is calibration and transportability: PRS performance often drops when applied across ancestries or clinical ascertainment regimes, so reporting ancestry composition, LD reference choice, and external validation performance is critical for clinical credibility.

**Figure 3:**
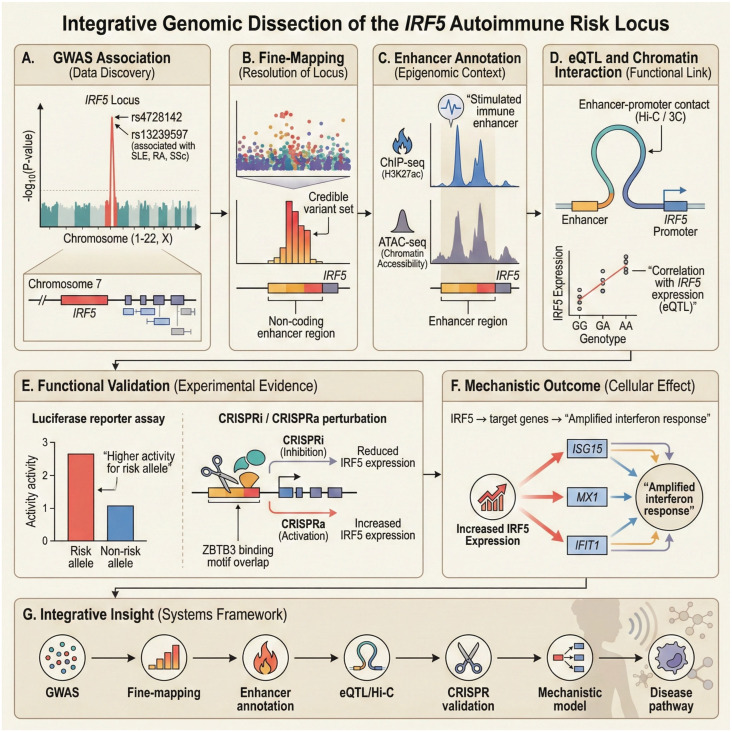
Illustrative integrative genomic dissection of the IRF5 autoimmune risk locus. Conceptual, literature-based schematic illustrating how the proposed integrative framework can be applied to interpret non-coding autoimmune GWAS signals, using the well-characterized IRF5 locus as an example. (**A**) GWAS association signal at the IRF5 locus linked to systemic lupus erythematosus, rheumatoid arthritis, or any other related autoimmune diseases. (**B**) Fine-mapping resolves the association to a credible set of non-coding variants localized within enhancer regions. (**C**) Immune-context-specific epigenomic annotation identifies stimulus-responsive enhancer activity overlapping prioritized variants. (**D**) Chromatin interaction and eQTL evidence link enhancer regions to IRF5 expression in activated immune cells. (**E**) Functional validation reported in the literature, including reporter assays and CRISPR-based perturbations, demonstrates allele-specific regulatory effects. (**F**) Increased IRF5 expression amplifies downstream interferon-stimulated gene programs. (**G**) Systems-level summary illustrating how sequential integration of genetic, epigenomic, and functional evidence supports mechanistic interpretation of non-coding risk. This figure synthesizes previously published findings and is intended solely as an illustrative example; no new experimental or analytical data are presented.

The IRF5 locus exemplifies how non-coding autoimmune risk can be resolved across genetic, epigenomic, and functional layers. This example synthesizes findings reported across multiple independent studies and demonstrates how statistical association can be translated into immune-context-specific mechanistic insight without generating new data. The workflow is summarized in Figure 3.

As illustrated in Figure 3, the integrative framework progresses from statistical association to mechanistic inference through a series of coordinated analytical steps. GWAS signals at the IRF5 locus [37] (Figure 3A) are first refined by fine-mapping to prioritize a credible set of non-coding variants [37,38] (Figure 3B). These variants are then evaluated in immune-context-specific epigenomic landscapes, revealing enhancer activity that emerges under inflammatory stimulation [38] (Figure 3C). Chromatin interaction data and state-dependent eQTL analyses link these enhancers to IRF5 transcription [37,38] (Figure 3D), while functional assays reported in the literature provide causal support for allele-specific regulatory effects [39,40] (Figure 3E). Elevated IRF5 expression amplifies interferon-responsive gene programs [41] (Figure 3F), culminating in a systems-level model that connects regulatory variation to autoimmune disease pathways [42] (Figure 3G).

Across the IRF5 locus, discordant signals from eQTL mapping, TWAS, and enhancer–gene linking reflect context-specific regulatory behavior rather than analytical failure. No single line of evidence is considered sufficient in isolation. Variants are prioritized for functional follow-up when multiple orthogonal signals converge within the same cell state or stimulation context, including GWAS colocalization, consistent regulatory directionality, and support from chromatin interaction or spatial localization. TWAS associations lacking colocalization or context-matched regulatory evidence are treated as provisional, while enhancer–gene links are weighted by cell-state specificity and activity. In this way, genetic, transcriptomic, and spatial data are integrated through a hierarchical, decision-oriented logic that emphasizes context, convergence, and mechanistic plausibility rather than nominal statistical significance alone.

Operationally, this framework treats variant prioritization as a cumulative inference problem, in which evidence strength increases with orthogonal concordance under matched immune contexts.

### 2.2. Single-Cell Resolution of Regulatory Mechanism

High-dimensional single-cell platforms now enable the dynamic modeling of gene regulation with unprecedented resolution, thereby overcoming the constraints of bulk profiling. Single-cell trajectory inference has enabled temporal ordering of immune activation states at high resolution [6]. This methodological shift revealed that over one-third of cis-eQTLs exhibit dynamic effect sizes, a regulatory dimension entirely missed in static-state designs. Concurrently, tensorQTL was employed for scalable and genotype-aware eQTL mapping across donor-specific pseudobulk transcriptomes, while mashR quantified the magnitude of shared versus condition-specific effects, refining signal detection across diverse cell states and activation stages.

Embedding eQTLs within coexpression modules links genetic effects to functional immune programs rather than isolated genes.

Shifts in cell composition across donors or stimulation time can confound dynamic eQTL detection if not explicitly modeled [43,44].

Modules such as GM2 (metabolic reprogramming), GM3 (cell cycle), and GM9 (immune effector processes) emerged as quantitative units linking variant effects to functional axes of T cell biology. Within this framework, the identification of subpopulation-specific eQTLs for CTLA4 and TYK2 serves not as isolated findings, but as proof-of-principle for how analytical modularity and temporal granularity converge to uncover actionable, context-specific regulatory mechanisms that would otherwise remain cryptic [6].

**Figure 4:**
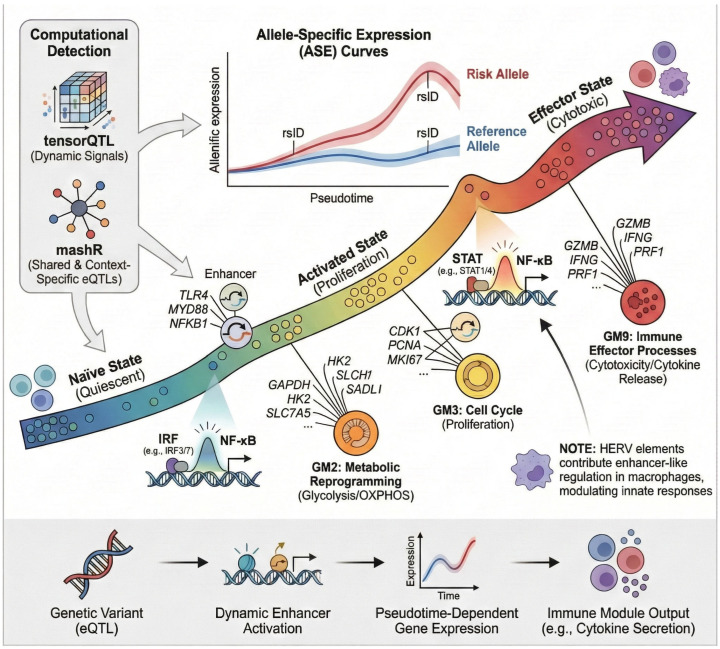
Dynamic genetic regulation across single-cell activation trajectories in the immune system. Schematic illustrating how single-cell transcriptomics resolves temporal and state-dependent genetic regulation. Continuous pseudotime trajectories of immune activation reveal dynamic allele-specific expression (ASE) of risk and reference alleles detected by tensorQTL and mashR. Variants modulate enhancer activity and transcription-factor binding (interferon regulatory factor, IRF; nuclear factor kappa B, NF-κB; signal transducer and activator of transcription, STAT), linking dynamic expression to gene modules (GM2, metabolic reprogramming via glycolysis/oxidative phosphorylation, OXPHOS; GM3, cell-cycle control; GM9, cytotoxic and cytokine effector processes). Human endogenous retroviral elements (HERVs) contribute enhancer-like regulation in macrophages, shaping innate immune responses. (Abbreviations: eQTL, expression quantitative trait locus; ASE, allele-specific expression; OXPHOS, oxidative phosphorylation; HERV, human endogenous retrovirus.). Genes, transcription factors, pathways, and gene modules shown are representative, literature-supported examples intended to illustrate general principles of dynamic genetic regulation rather than a locus-, gene-, or dataset-specific mechanism.

From a mechanistic perspective, dynamic eQTLs reveal that genetic effects are frequently gated by cellular activation trajectories rather than fixed cell identities [45]. Variants influencing transcription factors such as IRF, NF-κB, or STAT family members often exhibit maximal regulatory impact at transition points—during T-cell activation, B-cell differentiation, or macrophage polarization—when chromatin landscapes are permissive [46,47] (Figure 4).

Figure 4 illustrates how genotype-dependent enhancer activity evolves along a continuous pseudotime trajectory, coupling transcription-factor binding and enhancer activation to functional gene modules governing metabolic reprogramming, proliferation, and immune effector responses. This dynamic view underscores that autoimmune risk is encoded not only in which genes are affected but also in when and where regulatory control is exerted during immune differentiation. In practical terms, this motivates sampling strategies that deliberately capture transition states (early activation, lineage bifurcation points, and tissue-entry programs), because the strongest genotype-by-state interactions can be missed if profiling focuses only on canonical resting subsets.

While landmark autoimmune-focused studies have highlighted dynamic and state-dependent eQTLs [6,45] and multimodal chromatin expression regulation [48], regulatory variation more broadly propagates across multiple molecular layers. An integrative triangulation strategy, in which genetic association, regulatory activity, and gene expression evidence converge on the same locus, provides stronger mechanistic support than any single modality alone [49].

State-dependent single-cell eQTL (sc-eQTL) analyses reveal that approximately one-third of genetic variants exert their effects only within specific, continuously defined cell states, such as cytotoxic or regulatory memory T cell programs. This highlights why certain non-coding disease-associated alleles appear silent in aggregated or resting cell populations but become pathogenic under specific functional or immune-activated conditions [45]. This also implies an interpretive caution: “no eQTL in bulk” does not imply “no regulatory effect,” but may indicate that the relevant state is rare, transient, or tissue-restricted—strengthening the rationale for state-aware and tissue-aware mapping.

### 2.3. Shared and Disease-Specific Immune Modules

High-throughput transcriptomic profiling has enabled the classification of SLE patients into IFN-high and IFN-low molecular subtypes, with clinical correlations to renal pathology and serologic features such as anti-dsDNA and anti-Ro/SSA antibodies.

This stratification approach, initially established in early blood-based transcriptomic studies [50,51], has since been extended across platforms and tissues, linking interferon activation to clinical severity and organ involvement, including proliferative lupus nephritis [52,53].

Beyond interferon-centered modules, high-dimensional analyses of adaptive immune subsets add further resolution: spectral flow cytometry in SLE highlights expansion of activated naïve (aNAV) and age-associated double-negative B-cell subsets (DN2/DN3) B cells, with aNAV cells enriched for autoreactivity to dsDNA, correlating with both lupus nephritis and disease activity indices [54]. Similarly, single-cell transcriptomic and spatial profiling in RA synovium uncovers functional heterogeneity within peripheral helper T cells (Tph), distinguishing LAG3^−^ subsets that drive B-cell help from exhausted LAG3^+^ subsets [54].

To resolve the cellular contributors to ectopic B-cell help in early rheumatoid arthritis (RA), Murray-Brown et al. (2022) applied an integrative analytical workflow combining Opal-based multiplex immunofluorescence imaging, viSNE dimensionality reduction, and nearest-neighbor spatial modeling [55]. This multimodal pipeline identified PD-1^hi^ CXCR5^−^ T peripheral helper (Tph) cells as the predominant CD4^+^ T cell subset infiltrating synovial tissue in treatment-naïve early RA, with a marked 10-fold enrichment over classical T follicular helper (Tfh) cells. Quantitative spatial proximity analysis revealed that Tph cells localized within ~20 µm of both B cells and germinal center B cells, suggesting early synovial establishment of cognate T–B cell interactions that may initiate autoreactive maturation. These insights support Tph-driven B-cell activation circuits as a molecular hallmark of RA pathogenesis at disease onset [55]. From a regulatory-variant perspective, such modules become especially informative when paired with cell-state-specific regulatory maps (ATAC peaks, enhancer–gene links, and eQTL effects) that point to upstream “module controllers” (e.g., TF programs or costimulatory pathways) rather than only listing marker genes.

These complementary analytical insights highlight how modular frameworks extend across both interferon-driven and lymphocyte-intrinsic axes, providing deployable readouts for stratification/PD monitoring and nominating pathway-selective interventions (e.g., IFNAR/TYK2/JAK inhibitors; PD-1 pathway agonism or synovium-targeted bispecific delivery in early RA) [54,55].

**Figure 5:**
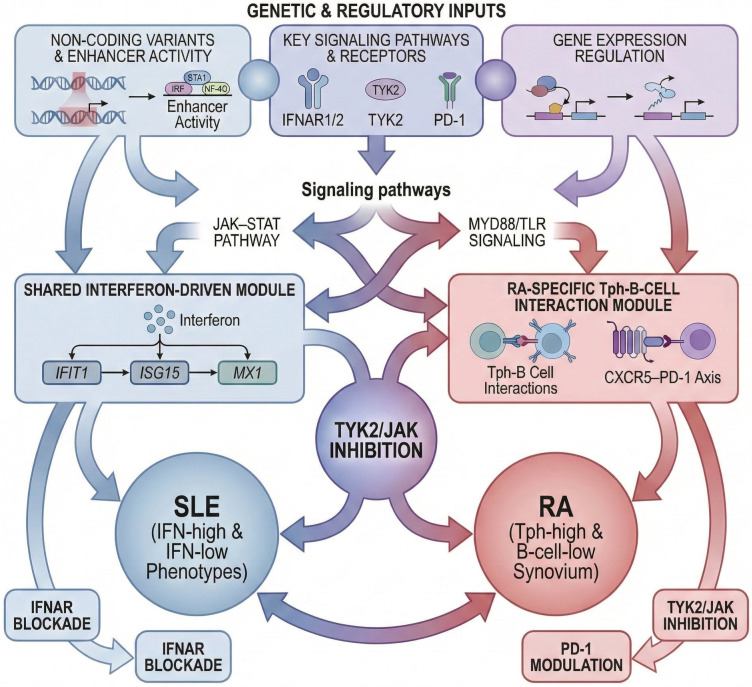
Systems-level framework linking genetic regulation to shared and disease-specific immune modules. Schematic model showing how non-coding regulatory variants and signaling pathways (JAK–STAT, MYD88/TLR) converge on shared interferon-driven and RA-specific B-cell/T-cell interaction modules. Interferon-stimulated genes (IFIT1, ISG15, MX1) define SLE endotypes, while the CXCR5–PD-1 axis characterizes T-peripheral helper (Tph)–B-cell interactions in RA. Central TYK2/JAK signaling represents a shared therapeutic node, with IFNAR blockade and PD-1 modulation acting as disease-selective interventions. Genes, pathways, immune modules, and therapeutic nodes shown are representative, literature-supported examples intended to illustrate systems-level regulatory logic rather than exhaustive or disease-specific causal relationships.

Collectively, these findings underscore that autoimmune endotypes arise from convergent regulatory logic rather than isolated molecular signatures. Figure 5 summarizes this systems-level framework, integrating upstream enhancer activity and key signaling nodes (JAK–STAT, MYD88/TLR) with shared interferon-driven and disease-specific B-cell/T-cell interaction modules. This mechanistic synthesis illustrates how genetic regulation, immune-cell circuitry, and therapeutic targeting intersect to define the phenotypic spectrum of SLE and RA.

Importantly, immune modules gain mechanistic relevance when their activity can be traced back to upstream regulatory variation. For example, interferon-driven modules are enriched for genetic variants affecting IRF binding sites, nucleic acid-sensing pathways, and negative feedback regulators [56,57], while B-cell-centric modules often intersect variants influencing enhancer activity at immunoglobulin loci or costimulatory genes [58]. Linking modules to their regulatory origins transforms them from descriptive signatures into biologically grounded mediators of genetic risk.

In practice, this can be operationalized by testing whether module “hub genes” (high connectivity drivers) harbor colocalized eQTL/GWAS signals in the relevant cell state, and whether the inferred direction of effect matches the observed module activation (e.g., risk alleles increasing inducible ISG programs under IFN stimulation).

This modularity, mapped via transcriptomic and pathway deconvolution, accommodates disease-specific downstream effectors while supporting shared therapeutic strategies, including IFNAR blockade and TYK2/JAK inhibition.

## 3. Emerging Analytical Frontiers in Autoimmune Disease Research

### 3.1. Cell-State and Spatial Profiling Approaches

Resolution of context-specific immune modules has accelerated adoption of single-cell multiome and spatial profiling technologies. Single-cell multiome sequencing integrates RNA-seq and ATAC-seq to map gene expression and chromatin accessibility in the same cell, uncovering cell-state-specific regulatory elements that bulk assays overlook [48]. In RA synovium, this approach revealed dynamic chromatin peaks enriched for autoimmune GWAS heritability, implicating Tph cells, Tregs, and IFN-stimulated myeloid cells in pathogenesis. These findings clarify how noncoding variants exert effects in specific immune subpopulations [48]. A key mechanistic advantage of multiome data is that it can connect risk-variant-tagged regulatory elements (accessible peaks) to the transcriptional programs they control in the same cell state, thereby reducing ambiguity in enhancer-to-gene assignment that arises when chromatin and expression are profiled in separate samples.

Deconvolution frameworks such as cell2location [59], Tangram [60], and DestVI [61] enable integration of scRNA-seq with spatial transcriptomics, mapping single-cell states back into tissue coordinates. These inferred spatial maps provide the basis for downstream analyses of immune–stromal niches and potential cell–cell interactions, thereby contextualizing how noncoding risk variants exert their effects within tissue microenvironments. Coupled with dynamic trajectory inference using RNA velocity (scVelo) [62] and lineage priors, such approaches reconstruct transient regulatory programs and activation trajectories in synovial or renal microenvironments.

Spatial transcriptomics restores tissue context lost during dissociation, enabling the identification of immune–stromal niches that drive localized inflammation and tissue damage [63]. By resolving whether genetically implicated programs operate in infiltrating immune cells, resident stromal compartments, or both, spatial atlases provide critical insight into tissue-specific disease mechanisms and therapeutic targeting strategies.

At the tissue level, spatial organization functions as a regulatory layer that shapes immune activation by constraining cell–cell interactions and signaling thresholds [64].

The proximity of immune cells to stromal or parenchymal compartments influences cytokine exposure, antigen availability, and costimulatory signaling, thereby modulating the persistence and resolution of inflammation [64,65,66]. Non-coding variants that affect chemokine or adhesion programs can consequently alter immune cell recruitment, retention, or positioning within inflamed tissues, shifting pathogenic responses independently of intrinsic immune cell function [67,68]. This spatial regulation provides a mechanistic link between regulatory variation and tissue-specific disease manifestations, with direct implications for therapeutic targeting and patient stratification [69,70,71].

To support platform selection for autoimmune-focused spatial studies, Table 2 summarizes the key features, resolution, integration tools, and quality control metrics of major spatial transcriptomics platforms validated in immune and FFPE tissues.

For translation, platform choice determines whether spatial signals are robust in clinically available specimens (notably FFPE) and whether inferred niches can be linked to actionable biomarkers or treatment-relevant microenvironments.

### 3.2. Mapping Autoreactive Clones and Neoantigen Targets

TCR sequencing reveals that HLA polymorphisms shape TCR repertoires, creating public CDR3 motifs associated with disease [78]. Antigen-specific T cells—rare and phenotypically ambiguous—can now be isolated using peptide–MHC tetramers, enabling high-resolution phenotyping. In celiac disease, this has helped to identify pathogenic Th1/Tfh hybrids. In autoimmune genetics, this layer is directly relevant to non-coding regulation because risk variants can shape antigen presentation programs through effects on HLA expression and interferon-driven MHC upregulation [79]. In addition, regulatory variation can alter costimulatory and activation thresholds that govern clonal expansion, as well as chemokine or adhesion molecule expression that determines whether autoreactive clones enter, localize within, and persist in inflamed tissue niches [80,81]. Framing clonotype-level findings within this regulatory logic strengthens the mechanistic link between inherited variation, immune context, and selective expansion of pathogenic T-cell populations. Peptide–MHC tetramer approaches are most informative when paired with single-cell profiling, enabling antigen specificity and immune state to be resolved within the same cells.

Neoantigen mapping using mass spectrometry-based immunopeptidomics has uncovered microbiota-induced modifications, such as cysteine carboxyethylation, that create self-peptides recognized by autoreactive T cells [82].

A practical limitation is that immunopeptidomics depth is constrained by sample quantity, peptide abundance, and the search-space inflation introduced by post-translational modifications; therefore, studies often benefit from prioritizing candidate PTMs and integrating peptide evidence with TCR specificity (tetramers) or functional readouts (cytokine production, activation markers) [83].

In the context of regulatory variants, a useful synthesis is to ask whether risk alleles increase the probability of presenting (or responding to) modified peptides by upregulating antigen-processing pathways under stimulation, thereby converting an environmental exposure into sustained clonal activation.

### 3.3. Integrating Multi-Omics with Predictive Modeling

The diagnostic complexity of autoimmune diseases (AIDs), arising from phenotypic overlap and immune heterogeneity, has increased interest in systems-level frameworks that unify molecular, serologic, and clinical data [84]. Recent machine learning pipelines now transcend siloed analyses by integrating multi-modal omics (genomics, immunomics, metabolomics) with laboratory values and structured clinical features. Such integrative models have demonstrated predictive accuracies reaching 96% for disease classification tasks involving RA, SLE, and healthy controls, even in the presence of modest data imbalance [84].

Because very high accuracy can sometimes reflect cohort structure (batch effects, platform differences, treatment separation) rather than generalizable disease biology, it is important to report the evaluation design explicitly (e.g., nested cross-validation, batch-aware splits, external cohort validation) and to include calibration/clinical utility metrics where possible.

From a mechanistic standpoint, machine learning models are most informative when they recover immune pathways already implicated by genetic and functional evidence, rather than when they rely on diffuse feature combinations [85]. Models that prioritize interferon signaling, B-cell activation, or metabolic reprogramming align more closely with known autoimmune biology [86], whereas high accuracy driven by composite inflammatory markers may reflect disease activity rather than causation [87]. Thus, integration with genetic priors and perturbational data is essential to constrain predictive models within biologically meaningful solution spaces. In practical terms, this means explicitly testing whether top-ranked features map onto genetically supported modules (e.g., GWAS-enriched pathways or colocalized regulatory targets) and whether model explanations remain stable across resampling and cohorts—two conditions that strengthen interpretability claims.

A recent integrative pipeline by Kruta et al. (2024) exemplifies this approach, harmonizing diverse datasets through standardized preprocessing, accumulation-based binary encoding, and dimensionality reduction [84]. This yielded an 84-feature matrix spanning five data types. Notably, metabolomics showed the highest discriminative power for RA, while immunomics (e.g., IGHV4-34, CDR3 clone architecture) was enriched in SLE.

These findings underscore a shift from linear biomarker pipelines toward modular, high-dimensional analytics that preserve mechanistic insight. Such frameworks enable interpretable, precision diagnostics and offer a scalable foundation for clinical decision support systems.

## 4. Toward Precision Stratification and Translational Application

**Figure 6:**
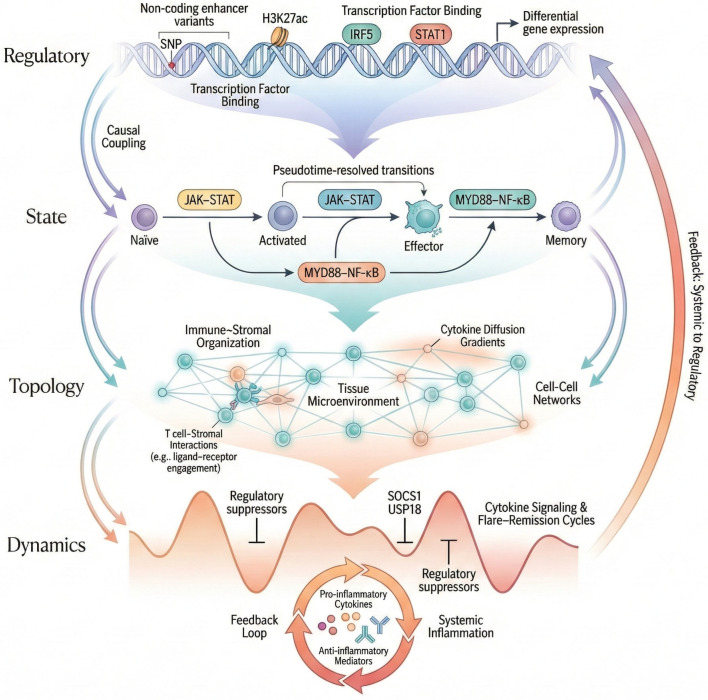
Regulatory–State–Topology–Dynamics (ReST-D) control landscape for precision autoimmunity. Conceptual model illustrating how autoimmune regulation arises from four interconnected layers. Genetic and epigenetic variants modulate transcriptional control and chromatin accessibility (Regulatory; for example, IRF- or STAT-mediated enhancer activity), shaping immune-cell activation and differentiation programs (State). These transitions reorganize immune–stromal interactions and cytokine gradients within tissue microenvironments (Topology). Temporal feedback loops governing cytokine flux and resolution (Dynamics) complete a multi-scale control system linking molecular regulation to clinical heterogeneity. Genes, signaling pathways, regulatory factors, and feedback mechanisms shown are representative, literature-supported examples intended to illustrate the ReST-D conceptual framework rather than exhaustive or disease-specific causal models.

The convergence of multi-omics, single-cell, and spatial technologies has transformed our ability to translate mechanistic discoveries into clinically meaningful frameworks. As summarized in Figure 6, autoimmune disease can be conceptualized as the product of interconnected regulatory layers, spanning chromatin-level control of gene expression, cell-state transitions, tissue organization, and cytokine-mediated feedback loops.

This integrative perspective provides the conceptual foundation for precision stratification, in which patients are classified not solely by clinical presentation but by shared molecular circuits and regulatory dynamics. Mechanistic insights from these layers now underpin emerging approaches in analytical endotyping, predictive modeling, and network-based drug repurposing, defining a continuum from systems-level understanding to individualized therapeutic application.

### 4.1. Patient Stratification and Analytical Endotyping

As multi-omics frameworks evolve to capture genetic, epigenetic, and transcriptional complexity across cell states, they increasingly offer not just mechanistic insights but clinically meaningful axes of variation. One critical application of this analytical depth is the identification of molecular endotypes—disease subgroups defined by shared regulatory programs, cell-type compositions, and dynamic immune states. Single-cell and mass-cytometry profiling of RA synovium has revealed fibroblast and monocyte subclusters (e.g., THY1^+^ HLA-DRA^+^ fibroblasts, IL-1β^+^ monocytes) associated with resistance to therapy [88]. In pediatric lupus, longitudinal transcriptomic profiling has uncovered immune signatures predictive of nephritis progression and treatment response [89]. Similar latent-state models have shown value across other immune-mediated contexts, including neuroinflammation and transplant rejection, where disease trajectory depends on immune state rather than fixed diagnosis. Extending these approaches to cross-cohort, multi-modal datasets may uncover shared axes of stratification that unify disease mechanisms across phenotypes.

These examples illustrate how unsupervised clustering and latent factor modeling—including methods like Multi-Omics Factor Analysis (MOFA)—can resolve patient-level heterogeneity into interpretable, stratifiable patterns with direct implications for diagnostics and intervention [90].

### 4.2. Predictive Modeling of Flares and Progression

Predictive modeling of autoimmune flares differs fundamentally from static disease classification, as flares are episodic, heterogeneous, and strongly influenced by immune state, treatment exposure, and tissue context [91]. Clinically relevant prediction targets include flare onset, severity, and near-term risk windows rather than binary disease status [92]. These outcomes are challenging to model because molecular changes often precede clinical manifestations by variable and disease-specific intervals, and immune signatures may be transient, treatment-modulated, or tissue-restricted [93].

Importantly, many predictive models rediscover established inflammatory programs, including JAK–STAT and interferon-γ signaling, highlighting their robustness across analytical paradigms [94]. In translational contexts, however, overall accuracy or AUC alone can be misleading, particularly in imbalanced datasets where disease flares or severe outcomes are infrequent [95]. Positive and negative predictive values are therefore critical for evaluating clinical utility, as they determine the reliability of predicted high-risk and low-risk states in real-world populations [96]. Models that achieve high negative predictive value may be especially valuable for ruling out imminent flares, whereas high positive predictive value is essential when predictions are intended to trigger treatment escalation or invasive monitoring [97].

Anticipating autoimmune flares remains a clinical priority. In RA, ensemble machine learning models trained on tapering data (RETRO trial) predicted flares with AUC ~0.81, identifying biologic dose change, Disease Activity Score-28 (DAS-28), and inflammatory markers as top features [98]. Similarly, genetic progression scores combining polygenic risk with EHR-linked genetics stratified preclinical SLE and RA risk [99]. Integration of longitudinal clinical and molecular data enables individualized flare-risk estimation and treatment adjustment.

Predictive accuracy alone does not establish clinical utility, particularly in imbalanced autoimmune datasets. Models gain robustness and interpretability when predictive features are constrained by mechanistically supported immune programs, such as interferon module dynamics, cell-state transitions, or genetically anchored regulatory pathways [100]. Anchoring predictive models to regulatory genetics and immune context improves stability across cohorts, facilitates biological interpretation, and enables outputs to be mapped onto actionable clinical decision points, including monitoring intensity, treatment adjustment, or flare-prevention strategies [101].

When integrated with the modular omics approaches discussed earlier, these models offer a scalable route toward proactive, precision-guided care.

### 4.3. AI-Guided Drug Repurposing and Network-Based Target Discovery

Beyond diagnostics, artificial intelligence increasingly supports therapeutic innovation. Machine learning frameworks and network-based analyses have demonstrated strong predictive performance in identifying repurposable drugs and novel molecular targets. For instance, AI-guided transcriptomic and signaling network models have elucidated key inflammatory pathways (e.g., JAK–STAT, IFN-γ) in autoimmune skin disorders such as psoriasis, suggesting new drug repurposing opportunities [102]. Similarly, machine learning frameworks integrating large-scale biomedical datasets have accelerated drug repurposing efforts across diverse diseases [103], while network pharmacology approaches have revealed novel therapeutic candidates for autoimmune disorders [104].

Network-based tools such as the DIME platform integrate immune cel-specific transcriptomes with disease–gene–drug interactions, nominating unexpected targets (e.g., lifitegrast for Crohn’s disease) [105]. These approaches exemplify how regulatory modules identified in omics data can be layered with pharmacogenomic networks to accelerate drug repurposing and target prioritization—extending the utility of analytical logic beyond biomarker discovery. Crucially, the most informative repurposing frameworks do not rely on prediction accuracy alone, but on whether nominated drugs intersect genetically and mechanistically supported disease modules. By anchoring AI-driven inference to regulatory circuits implicated by GWAS, eQTL/TWAS, and single-cell analyses, these approaches prioritize compounds that are predicted to modulate upstream drivers of immune dysregulation rather than downstream inflammatory readouts.

A key advance of intersecting drug signatures with genetically and mechanistically supported disease modules is the transition from associative repurposing toward causal prioritization [106]. Machine learning-driven integration of GWAS loci with eQTL, TWAS, and epigenomic datasets enables causal prioritization of disease-associated regulatory genes. By mapping these features within cell-type-specific regulatory landscapes, AI frameworks can distinguish upstream, genetically driven mechanisms of immune dysregulation from secondary inflammatory signals—enhancing confidence in therapeutic target selection and drug repurposing [107].

This integrative intersection enables the stratification of repurposing candidates by both mechanistic relevance and genetic support, reducing false-positive nominations and facilitating rational selection of drugs most likely to modify disease trajectory [108]. In this framework, AI does not merely rediscover known pathways but contextualizes them within genetically constrained immune circuits, yielding actionable hypotheses for precision therapeutic targeting.

Despite their promise, AI-guided repurposing frameworks introduce limitations that must be addressed for clinical credibility. Black-box models that prioritize prediction accuracy without mechanistic interpretability can obscure causal reasoning, complicating biological validation and regulatory acceptance [109,110,111,112]. In addition, training data are often skewed toward well-studied diseases and tissue contexts, as widely documented across biomedical and precision-medicine research [113,114,115,116], with similar limitations noted in recent applied AI studies [117,118,119]. Privacy constraints further limit access to large, harmonized clinical–omics datasets [120,121], motivating the use of federated or summary-level learning approaches [122,123]. These challenges reinforce the need for transparent, mechanism-aware models in which AI acts as a constrained inference layer, guided by regulatory genetics and immune context, rather than an unconstrained predictor of therapeutic relevance.

### 4.4. Generalizability of the Framework Across Autoimmune Diseases

Although this review mainly emphasizes SLE and RA, this focus reflects the depth and maturity of available integrative genomic, single-cell, spatial, and functional datasets in these diseases rather than a conceptual limitation of the proposed framework.

SLE and RA currently provide the most complete examples in which non-coding regulatory variants have been traced across genetic association, immune context, and functional validation, enabling clear illustration of analytical logic. They are therefore used as representative exemplars rather than exclusive disease targets.

**Figure 7:**
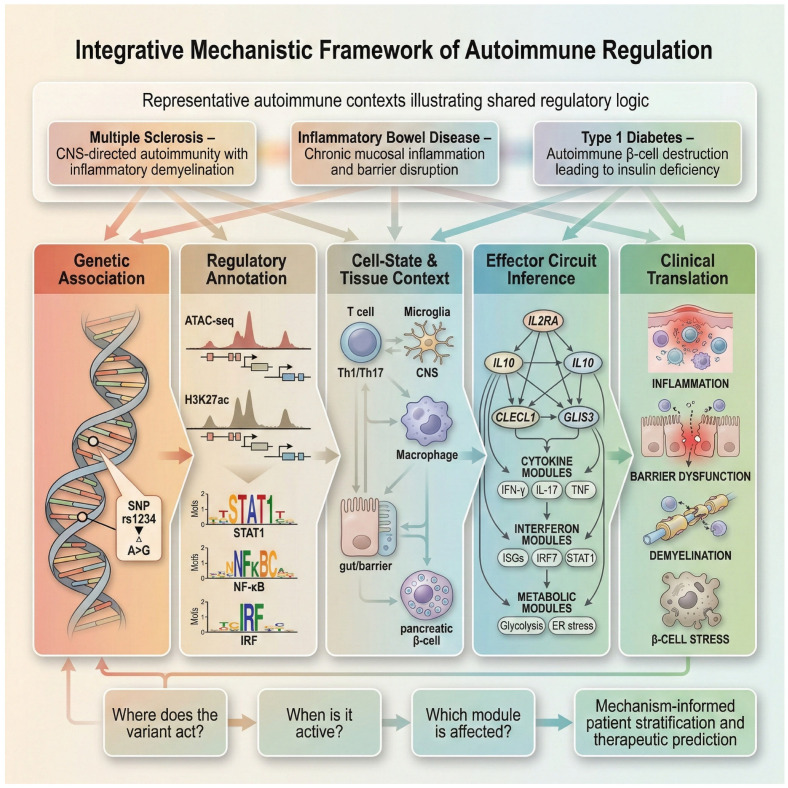
Conceptual generalization of the integrative framework beyond SLE and RA. This schematic illustrates, in conceptual form, how the analytical framework described in this review extends across autoimmune contexts without implying shared molecular mechanisms. Top: representative examples—multiple sclerosis, inflammatory bowel disease, and type 1 diabetes—highlight distinct tissue settings in which context-dependent regulatory variation operates. Middle: the framework links genetic association to regulatory annotation and cell-state resolution, showing how non-coding variants modulate enhancer activity and transcription-factor binding (for example, STAT1, NF-κB, IRF) within relevant immune or tissue cells such as T cells, macrophages, microglia, epithelial cells, or pancreatic β-cells. Integrating chromatin and single-cell datasets enables inference of representative effector genes (IL2RA, IL10, CLECL1, GLIS3) regulating cytokine, interferon, or metabolic programs. Bottom: decision nodes summarize analytical progression—determining where a variant acts, when it is active, and which effector process it influences—illustrating a transferable logic for mechanistic interpretation, patient stratification, and therapeutic hypothesis generation across autoimmune diseases.

Importantly, the regulatory principles described throughout this review extend across a broad range of autoimmune diseases in which genetic risk is mediated by context-dependent immune regulation. Figure 7 provides a conceptual overview of how the proposed integrative framework generalizes beyond SLE and RA, illustrating shared regulatory logic across multiple sclerosis, inflammatory bowel disease, and type 1 diabetes rather than disease-specific mechanisms.

In multiple sclerosis, non-coding risk variants are enriched in enhancers active in stimulated T cells and microglia, with state-dependent eQTLs implicating interferon and antigen-presentation pathways [124,125].

In inflammatory bowel disease, fine-mapped GWAS loci intersect epithelial- and myeloid-specific regulatory elements, with chromatin and eQTL analyses linking non-coding variants to cytokine and barrier-function genes in inflamed tissue [126,127].

In type 1 diabetes, age-stratified genetic analyses reveal that risk variants with stronger effects in early-onset cases (<7 years) localize near genes functioning in both pancreatic β-cells (e.g., GLIS3) and immune pathways (e.g., IL2RA, IL10, IKZF3, THEMIS, CTSH). These findings indicate that the most aggressive, early-diagnosed forms of the disease arise from combined dysregulation of β-cell stress sensitivity and T- and B-cell activation and selection processes [128].

Across these diseases, the same analytical sequence recurs: statistical association is refined by regulatory annotation, immune-state resolution identifies when and where variants act, and integrative eQTL, chromatin, and perturbational evidence nominates effector genes or regulatory modules. Thus, the proposed framework generalizes not by disease-specific signatures, but by a shared regulatory logic governing how non-coding variants shape immune responses under defined contexts.

From a translational perspective, this generalizability is operationalized through decision points rather than disease labels. These include identifying the immune cell state or tissue niche in which a variant is functional, establishing regulatory directionality along a stimulus–response axis, and mapping variant effects onto effector modules linked to clinical outcomes such as flare risk, treatment response, or therapeutic vulnerability. These steps can be implemented using disease-appropriate datasets without altering the underlying analytical architecture.

Accordingly, the translational value of this framework lies in enabling mechanism-informed patient stratification and target prioritization across autoimmune phenotypes, rather than in cataloging disease-specific markers. By anchoring predictive modeling, endotyping, and drug repurposing to genetically and mechanistically supported regulatory circuits, the approach provides a scalable foundation for precision immunology beyond SLE and RA.

## 5. Technical Challenges in Translating Analytical Frameworks

Translating analytical frameworks into clinical pipelines faces several methodological bottlenecks. Autoimmune flares exhibit irregular dynamics poorly captured by models assuming periodicity. Time-series methods accommodating stochastic inputs and external triggers are essential for accurate forecasting [129]. Additionally, real-world omics datasets suffer from non-random missingness and batch effects. Discarding incomplete data introduces bias, while naïve imputation reduces fidelity—highlighting the need for structure-aware imputation techniques [130]. Class imbalance—such as infrequent flares versus remission—also skews model training. Methods such as Synthetic Minority Over-sampling Technique (SMOTE) and cost-sensitive learning help address imbalance but require careful calibration to avoid overfitting [131,132].

In autoimmune settings, additional domain-specific confounders can be decisive: immunosuppressive therapy (e.g., corticosteroids or biologics) can suppress interferon and activation signatures, shifting module scores independently of underlying pathogenesis [133]; tissue sampling heterogeneity (blood vs. synovium vs. kidney) can create apparent “missingness” that is biologically structured [134]; and flare labels may be noisy or delayed relative to molecular changes, complicating supervised learning [135]. Explicitly modeling treatment as a time-varying covariate and aligning molecular sampling to clinical event timing can reduce these biases. Addressing these issues will determine whether precision frameworks can scale beyond research settings into clinical decision support.

Beyond analytical and biological considerations, clinical deployment of PRS and multi-omics prediction models requires meeting explicit regulatory and validation standards. In most jurisdictions, tools that inform diagnosis, prognosis, or treatment decisions are subject to requirements for analytical validity (assay accuracy, reproducibility, and quality control), clinical validity (robust discrimination and calibration with external validation in representative cohorts), and clinical utility (evidence that model outputs improve clinical decision-making or patient outcomes) [136,137]. For PRS, additional constraints include ancestry portability, transparent reporting of variant composition and linkage disequilibrium references, and clinically interpretable thresholds linked to defined management actions. These factors are critical for clinical reliability and equitable deployment.

Firstly, the ancestry portability must be explicitly addressed, as PRS derived primarily from European-ancestry cohorts perform substantially worse in non-European populations due to differences in allele frequencies, LD patterns, and effect-size transferability [138,139]. Improving trans-ancestry portability requires incorporating diverse training data, functional variant prioritization, and ancestry-specific LD reference panels to ensure robust performance across populations. Also, transparent reporting of variant composition, LD reference datasets, model-building methods, and performance metrics across ancestries is essential for reproducibility and interpretability [140,141].

Finally, clinically interpretable thresholds must be defined and linked to evidence-based management actions, as ancestry-specific PRS have been shown to enhance clinical decision-making—for example, reclassifying patients at borderline cardiovascular risk and refining preventive strategies [142]. Together, these principles support transparent, equitable, and clinically actionable implementation of PRS in precision medicine.

For multi-omics and machine learning models, clinical credibility depends on rigorous control of technical and methodological variability. Standardization of pre-analytical workflows, including sample collection, handling, and data acquisition, is essential to ensure comparability and reproducibility across laboratories and sites, as emphasized in international clinical omics frameworks [143], and in multicenter data harmonization studies [144].

Correction and monitoring of batch effects are also critical because technical variation between instruments, centers, or acquisition periods can obscure true biological signals. Systematic assessment and mitigation of such batch effects are now recognized as fundamental to the reliability of omics-based studies [145]. For machine learning-based models, credibility depends on prospective evaluation and post-deployment monitoring to identify performance drift as data distributions and clinical practices evolve over time [146,147]. Accordingly, the translation from research to clinical application should proceed through a staged validation process. This process begins with retrospective model training and internal cross-validation, followed by replication across multiple independent cohorts to assess generalizability [148], and culminates, when possible, in prospective or pragmatic clinical evaluation to establish real-world performance and clinical benefit [149].

Meeting these regulatory and validation requirements further depends on supporting computational and reporting infrastructure that enables reproducibility, validation, and clinical portability. Supporting infrastructure is also considered, including standards and workflows that improve reproducibility and clinical portability. Findable, Accessible, Interoperable and Reusable (FAIR)/Global Alliance for Genomics and Health (GA4GH)-aligned data schemas, integration with clinical records through Health Level Seven Fast Healthcare Interoperability Resources (HL7 FHIR), and containerized workflows can mitigate irreproducibility and facilitate external validation [84,150,151].

## 6. Conclusions

Current limitations in interpreting non-coding autoimmune risk arise primarily from static annotation and under-resolved immune-state modeling. Overcoming these barriers will likely require modular, context-aware frameworks that integrate functional genomic assays, cell-state-specific regulatory mapping, and spatially resolved transcriptomics. Such approaches allow immune regulation to be modeled as a dynamic, multidimensional process, rather than a static signature.

A key contribution of this review is the integration of these advances into a stepwise interpretive architecture that progresses from statistical prioritization to immune-context resolution and, where available, perturbational validation—thereby clarifying which non-coding associations are supported by regulatory directionality and which remain probabilistic.

Importantly, the translational value of these approaches does not derive from any single method, but from their coordinated application within context-aware analytical frameworks. Evidence across recent studies indicates that integrating multi-omics, single-cell, and machine learning approaches can enable the definition of actionable disease endotypes, support predictive modeling of disease course, and inform therapeutic prioritization. Collectively, this integrative logic provides a practical pathway for translating complex immunogenomic data into stratified care in systemic autoimmune diseases.

## Figures and Tables

**Table 1:** Summary of major TWAS models, including their expression references, use of regulatory priors, validation metrics, post-analysis tests, key limitations, and reporting requirements.

Model	Expression Reference	Regulatory Priors	Cross-Validated R^2^	Post-Analysis Tests	Key Limitations	Reporting Checklist	Reference
FUSION	GTEx v7/v8 (bulk, tissue-specific)	None	Per-gene 5-fold CV-R^2^ from GTEx panel; often <0.2 and gene/tissue-specific	Coloc/SMR-HEIDI (optional)	LD reference mismatch; tissue heterogeneity	GTEx panel version; LD reference; CV scheme; FDR control	[28]
S-PrediXcan/PrediXcan	GTEx v7/v8 (bulk, tissue-specific)	None	Report per-gene CV-R^2^; avoid overinterpreting high train R^2^	Coloc/SMR (HEIDI)	Model portability across ancestry; LD dependency	Model source; CV-R^2^; FDR; software version; LD ref panel	[29]
TESLA	eQTL panels (bulk or pseudobulk; ancestry-matched)	Optimal linear weighting of ancestry-specific summary stats	Per-gene CV-R^2^ depends on the underlying eQTL models; TESLA is a meta-analysis stage method	Coloc/SMR/HEIDI as applicable	LD heterogeneity; ancestry mismatch	GWAS ancestry; eQTL panel details; CV-R^2^; meta-strategy	[30]
PUMICE	GTEx v7/v8, or pseudobulk from sorted/scRNA	Hi-C + ATAC-seq chromatin priors define cis windows	Higher per-gene CV-R^2^ than UTMOST/PrediXcan; validated across traits	Coloc (PP4 > 0.9) used for prioritization	Sensitive to choice of 3D priors; limited scRNA data	Expression model; chromatin prior type; CV-R^2^; colocalization metric	[31]
UTMOST	GTEx v6p/v7, multi-tissue expression panels	Shared effect modeling across tissues	CV-R^2^ computed per gene per tissue; benefits from multi-tissue correlation	Coloc/SMR/MAGMA compatible	Assumes cross-tissue effect sharing; lower per-tissue resolution	Tissue panel; model assumptions; LD panel; multi-testing correction	[32]
TIGAR-V2	GTEx v8	Bayesian DPR or Elastic-Net	Burden & variance components	Improved imputation R^2^ vs. PrediXcan	Coloc	Bayesian model interpretation complexity	[33]
MTWAS	GTEx, DICE, OneK1K	Cross-tissue vs. tissue-specific partitioning	Non-parametric TWAS stats	Better than PrediXcan	Coloc, replication	Computationally complex	[34]
A-TWAS	Multiple transcriptomic panels	Bayesian shrinkage (e.g., Horseshoe+)	ACAT-based omnibus *p*-values	Enhanced prediction R^2^	Coloc	Complexity in prior selection	[35]
EpiXcan	GTEx, STARNET	Epigenomic-informed (e.g., chromatin states)	Z-score/TWAS *p*-value	Trait-specific; improves CAD prediction	Coloc, CRISPR validation	Limited by prior availability, tissue bias	[36]

Abbreviations: TWAS—Transcriptome-Wide Association Study; GTEx—Genotype-Tissue Expression; CV-R^2^—Cross-validated Coefficient of Determination; LD—Linkage Disequilibrium; Coloc—Colocalization; SMR—Summary-based Mendelian Randomization; HEIDI—Heterogeneity in Dependent Instruments; FDR—False Discovery Rate; DPR—Dirichlet Process Regression; ACAT—Aggregated Cauchy Association Test; CAD—Coronary Artery Disease; scRNA—Single-cell RNA sequencing; Hi-C—Chromatin Conformation Capture; ATAC-seq—Assay for Transposase-Accessible Chromatin sequencing.

**Table 2:** Spatial transcriptomics platforms and analysis workflows for autoimmune tissues.

Platform	Resolution	Assay Scope	FFPE Support	Integration/Deconvolution	QC Metrics Reported	Citation
10× Visium (standard)	55 µm	Whole-transcriptome (probe panel)	Yes	Integrated with Xenium and Aspect Analytics for image integration.	Concordance with Xenium data; low signal in fibrotic FFPE tissues (kidney sample not satisfactory); pathologist region annotation (stroma, carcinoma, immune cells)	[72]
CosMx SMI	Subcellular (numeric value not specified in the text)	Targeted panel (CosMx 960 genes stated in Results; 1000-plex used in Methods: 950 core + 50 add-on)	Yes	AtoMx export, Seurat (SCTransform/UMAP), InSituType (annotations), Voyager (Moran’s I)	Lower sensitivity and dynamic range than Xenium (4.7× vs. 372× over controls); higher background; lower Moran’s I; better membrane-based segmentation; reproducible (r > 0.99); weak T-cell detection (62 vs. 1 cells > 5 transcripts).	[73]
Xenium	~0.5–1 µm	Targeted (up to 5000 genes)	Yes	Integrated with Visium, snRNA-seq	Background subtraction, spatial drift, signal intensity	[74]
10× Visium HD	~10 µm (single-cell-scale)	Whole-transcriptome (fresh frozen tissues)	No	Seurat, scRNA-seq alignment	Not specified; implied focus on clustering accuracy	[75]
Stereo-seq	0.5 µm (subcellular)	Whole-transcriptome (unbiased poly-dT capture)	No	Compared with CODEX proteomics and scRNA-seq ground truth datasets	Capture sensitivity; specificity; diffusion control; cell segmentation accuracy; cell annotation; spatial clustering; transcript–protein alignment with CODEX	[76]
Slide-seqV2	~10 µm	Whole-transcriptome	No	scRNA-seq trajectory tools, Tangram (common)	Bead registration accuracy, spatial resolution, RNA capture efficiency	[77]

Abbreviations: FFPE, formalin-fixed paraffin-embedded; scRNA-seq, single-cell RNA sequencing; snRNA-seq, single-nucleus RNA sequencing; CODEX, co-detection by indexing; QC, quality control.

## Data Availability

No new data were created or analyzed in this study.

## Abbreviations

10×	10× Genomics
3D	Three-dimensional
ABC	Activity-by-contact (enhancer–gene linking model)
ACAT	Aggregated Cauchy Association Test
AIDs	Autoimmune diseases
ANA	Antinuclear antibodies
anti-dsDNA	Anti–double-stranded DNA antibodies
anti-Ro/SSA	Anti-Ro/Sjögren’s-syndrome-related antigen A antibodies
ASE	Allele-specific expression
ATAC-seq	Assay for Transposase-Accessible Chromatin sequencing
AtoMx	CosMx analysis/export environment (NanoString)
AUC	Area under the curve
aNAV	Activated naïve (B-cell subset)
B cell/B-cell	B lymphocyte
β-cell	Pancreatic beta cell
caQTL	Chromatin accessibility quantitative trait locus
CAD	Coronary artery disease
CDR3	Complementarity-determining region 3
ChIP–qPCR	Chromatin immunoprecipitation followed by quantitative PCR
CLECL1	C-type lectin-like receptor 1 (gene symbol)
Coloc	Colocalization (genetic colocalization analysis)
CMap	Connectivity Map
CODEX	Co-detection by indexing
CRISPR	Clustered Regularly Interspaced Short Palindromic Repeats
CRISPRa	CRISPR activation
CRISPRi	CRISPR interference
CTSH	Cathepsin H (gene symbol)
CV-R^2^	Cross-validated coefficient of determination (R^2^)
DAS-28	Disease Activity Score in 28 joints
DestVI	Deep generative model for spatial transcriptomics deconvolution
DICE	Database of Immune Cell Expression (immune eQTL/resource panel)
DIME	Disease–Immune cell–Molecular mechanism–drug platform
DN2/DN3	Double-negative B-cell subsets 2 and 3
DNA	Deoxyribonucleic acid
DPR	Dirichlet Process Regression
eQTL	Expression quantitative trait locus
EHR	Electronic health record
Enrichr	Gene set enrichment tool/resource
EpiXcan	Epigenomic-informed TWAS framework
FAIR	Findable, Accessible, Interoperable, Reusable data principles
FDR	False discovery rate
FFPE	Formalin-fixed paraffin-embedded
FHIR	Fast Healthcare Interoperability Resources
FINEMAP	Fine-mapping method/software
FUSION	Functional Summary-based Imputation (TWAS framework)
GA4GH	Global Alliance for Genomics and Health
GLIS3	GLIS family zinc finger 3 (gene symbol)
GM2/GM3/GM9	Gene modules 2/3/9 (as defined in referenced study)
gProfiler2	Functional enrichment tool
GTEx	Genotype-Tissue Expression project
GWAS	Genome-wide association studies
H3K27ac	Histone H3 lysine 27 acetylation
HDAC	Histone deacetylase
HEIDI	Heterogeneity in Dependent Instruments (SMR follow-up test)
HERV/HERVs	Human endogenous retrovirus/elements
Hi-C	Chromatin conformation capture (Hi-C)
HL7	Health Level Seven
hQTL	Histone quantitative trait locus
IBD	Inflammatory bowel disease
IFN	Interferon
IFNAR	Interferon alpha/beta receptor
IFN-γ	Interferon gamma
IFIT1	Interferon-induced protein with tetratricopeptide repeats 1
IGHV4-34	Immunoglobulin heavy variable 4-34 (gene segment)
IKZF3	IKAROS family zinc finger 3 (gene symbol)
IL-1β	Interleukin 1 beta
IRF	Interferon regulatory factor (family)
IRF5	Interferon regulatory factor 5
ISG15	Interferon-stimulated gene 15
JAK–STAT	Janus kinase–signal transducer and activator of transcription
LAG3	Lymphocyte activation gene 3
LASSOSUM	LASSO summary statistics method
LD	Linkage disequilibrium
MAGMA	Multi-marker Analysis of GenoMic Annotation
MHC	Major histocompatibility complex
MOFA	Multi-Omics Factor Analysis
MPRA	Massively Parallel Reporter Assays
MS	Multiple sclerosis
MTAG	Multi-Trait Analysis of GWAS
MTWAS	Multi-tissue/multi-trait TWAS
MX1	MX dynamin-like GTPase 1
MYD88	Myeloid differentiation primary response 88
NF-κB	Nuclear factor kappa B
OneK1K	1000 Genomes reference panel shorthand
OXPHOS	Oxidative phosphorylation
PCA	Principal component analysis
PD-1	Programmed cell death protein 1
Pol II	RNA polymerase II
PP4	Posterior probability for hypothesis 4
pQTL	Protein quantitative trait locus
PPR	Posterior probability of replicability
PRS	Polygenic risk score
PTM/PTMs	Post-translational modification(s)
PUMICE	Prediction Using Models Informed by Chromatin conformations and Epigenomics
qPCR	Quantitative polymerase chain reaction
RA	Rheumatoid arthritis
RATES	Replicability Analysis of Trait-Associated Signals
ReST-D	Regulatory–State–Topology–Dynamics
RNA-seq	RNA sequencing
RNP	Ribonucleoprotein
sQTL	Splicing quantitative trait locus
sc-eQTL	Single-cell eQTL
scRNA-seq	Single-cell RNA sequencing
scVelo	Single-cell RNA velocity framework
Seurat	Single-cell analysis toolkit
SLE	Systemic lupus erythematosus
SMOTE	Synthetic Minority Over-sampling Technique
SMR	Summary-based Mendelian Randomization
SNP	Single-nucleotide polymorphism
SPATCH	Spatial transcriptomics alignment with multiplexed protein data
STAR-NET	STARNET tissue expression resource
STAT	Signal transducer and activator of transcription (family)
STAT1	Signal transducer and activator of transcription 1
SuSiE	Sum of Single Effects (fine-mapping method)
T1D	Type 1 diabetes
TCR	T-cell receptor
TESLA	Chromatin-informed TWAS model
Tfh	T follicular helper T cell
THEMIS	Thymocyte-expressed molecule involved in selection
THY1	Thy-1 cell surface antigen
TLR	Toll-like receptor
Tph	T peripheral helper T cell
Treg/Tregs	Regulatory T cell(s)
TWAS	Transcriptome-Wide Association Study
UBASH3A	Ubiquitin-associated and SH3 domain-containing A
UMAP	Uniform Manifold Approximation and Projection
UMI	Unique molecular identifier
UT-MOST/UTMOST	Unified Test for MOlecular SignaTures
viSNE	Visualization of t-distributed stochastic neighbor embedding
WTA	Whole-transcriptome assay
Xenium	10× Genomics Xenium spatial transcriptomics platform
